# Supplementary material for: Universal PCR and antibody testing demonstrate little to no transmission of SARS-CoV-2 in a rural community
Source: medRxiv. 2020 Aug 17:2020.08.15.20175786. Preprint. [Version 1] doi: 10.1101/2020.08.15.20175786 (PMC7444294; doi:10.1101/2020.08.15.20175786)
Supplement: 1 [file NIHPP2020.08.15.20175786-supplement-1.pdf]

medRxiv preprint doi: https://doi.org/10.1101/2020.08.15.20175786.this version posted August 17, 2020. The copyright holder for this preprint (which was not certified by peer review) is the author/funder, who has granted medRxiv a license to display the preprint in perpetuity.
It is made available under a CC-BY 4.0 International license .

APPENDIX 1

SUPPLEMENTARY METHODS

1A. Testing the upper bound on the true number of infections

To test the upper bound on the true number of infections (via PCR), we calculated the probability of observing x=0 cases, conditional on there being exactly K cases in our population of size N (of which we tested n). We assume that the sensitivity (Se) and specificity (Sp) of the diagnostic test are known and fixed at 80% and 100%, respectively. We assume that the n individuals were sampled at random and so are representative of the entire population N. We use the hypergeometric distribution since we have a finite population size so trials are not independent, and binomial distributions to account for Se and Sp.

$$\Pr(X = 0; K, N, n, Se, Sp) = \sum_{y=0}^{K} \frac{\binom{K}{y}\binom{N-K}{n-y}}{\binom{N}{n}} \cdot (1 - Se)^{y} \cdot Sp^{n-y}$$

Code available at:
https://github.com/EPPIcenter/bolinas-analysis/blob/master/1A_Figure_1.R.

1B. Estimation of PCR prevalence

We estimated PCR prevalence in two ways: first, by using the binomial distribution to model prevalence (p) and second, by using the hypergeometric distribution to model prevalence. The latter is more appropriate in this scenario since testing was performed on a large proportion of the total population (upwards of 80%), so the hypergeometric distribution will yield narrower estimates of uncertainty. We again assume that the Se and Sp of the diagnostic test are known and fixed at 80% and 100%, respectively. As the binomial distribution is more frequently used for prevalence estimation, we provide Stan code to implement both models.

$$x \sim \text{Hypergeometric}\Big(n, \big(p \cdot Se + (1-p) \cdot (1 - Sp)\big) \cdot N, N - \Big(\big(p \cdot Se + (1-p) \cdot (1-Sp)\big)\Big) \cdot N\Big)$$

$$x \sim \text{Binomial}\big(N, p \cdot Se + (1-p) \cdot (1 - Sp)\big)$$

Code available at:
https://github.com/EPPIcenter/bolinas-analysis/blob/master/1B.1_PCR_prevalence_HGM.R
https://github.com/EPPIcenter/bolinas-analysis/blob/master/1B.2_PCR_prevalence_binomial.R

2A. Estimation of seroprevalence (one test)

medRxiv preprint doi: https://doi.org/10.1101/2020.08.15.20175786.this version posted August 17, 2020. The copyright holder for this preprint (which was not certified by peer review) is the author/funder, who has granted medRxiv a license to display the preprint in perpetuity.
It is made available under a CC-BY 4.0 International license .

We used the binomial distribution to estimate seroprevalence (*p*) separately for each assay *j*. We use the binomial distribution, which will yield conservative intervals as compared to the hypergeometric distribution used as above. The difference from the estimation of PCR prevalence is that we also estimate *Se* and *Sp* of the assays using validation data. The positive predictive values (PPV) are calculated directly from the estimates.

$$x_j \sim \text{Binomial}\left(N, p_j \cdot Se_j + (1 - p_j) \cdot (1 - Sp_j)\right)$$

$$\text{Positive controls testing positive}_j \sim \text{Binomial}\left(\text{Positive controls}_j,\ Se_j\right)$$

$$\text{Negative controls testing negative}_j \sim \text{Binomial}\left(\text{Negative controls}_j,\ Sp_j\right)$$

$$\text{PPV}_j = \frac{Se_j \cdot p_j}{\left(Se_j \cdot p_j\right) + \left((1 - Sp_j) \cdot (1 - p_j)\right)}$$

Code available at:
https://github.com/EPPIcenter/bolinas-analysis/blob/master/2A_Ab_prevalence_separate_binomial.R

2B. Estimation of seroprevalence (two tests)

We extended the approach in 2A above to jointly model the results of both assays x_{jk}, using the multinomial distribution as the generalization of the binomial distribution and now estimating a single seroprevalence *p*. We necessarily assumed conditional independence between the two assays *j* and *k*. For samples that were only tested on one platform, we allowed them to contribute to the binomial likelihood for the platform that they were tested on. The positive predictive values, which are now based on the results of both assays, are calculated directly from the estimates.

$$\begin{pmatrix} x_{++} \\ x_{+-} \\ x_{-+} \\ x_{--} \end{pmatrix} \sim \text{Multinomial}\begin{pmatrix} p \cdot Se_1 \cdot Se_2 \ + \ (1-p) \cdot (1 - Sp_1) \cdot (1 - Sp_2) \\ p \cdot Se_1 \cdot (1 - Se_2) \ + \ (1-p) \cdot (1 - Sp_1) \cdot Sp_2 \\ p \cdot (1 - Se_1) \cdot Se_2 \ + \ (1-p) \cdot Sp_1 \cdot (1 - Sp_2) \\ p \cdot (1 - Se_1) \cdot (1 - Se_2) \ + \ (1-p) \cdot Sp_1 \cdot Sp_2 \end{pmatrix}$$

$$\text{Positive controls testing positive}_j \sim \text{Binomial}\left(\text{Positive controls}_j,\ Se_j\right)$$

$$\text{Negative controls testing negative}_j \sim \text{Binomial}\left(\text{Negative controls}_j,\ Sp_j\right)$$

$$\text{PPV}_{++} = \frac{Se_j \cdot Se_k \cdot p}{\left(Se_j \cdot Se_k \cdot p\right) + \left((1 - Sp_j) \cdot (1 - Sp_k) \cdot (1 - p)\right)}$$

medRxiv preprint doi: https://doi.org/10.1101/2020.08.15.20175786.this version posted August 17, 2020. The copyright holder for this preprint
(which was not certified by peer review) is the author/funder, who has granted medRxiv a license to display the preprint in perpetuity.
It is made available under a CC-BY 4.0 International license .

$$\mathrm{PPV}_{+-} = \frac{Se_j \cdot (1 - Se_k) \cdot p}{\left(Se_j \cdot (1 - Se_k) \cdot p\right) + \left(\left(1 - Sp_j\right) \cdot Sp_k \cdot (1 - p)\right)}$$

$$\mathrm{PPV}_{-+} = \frac{(1 - Se_j) \cdot Se_k \cdot p}{\left((1 - Se_j) \cdot Se_k \cdot p\right) + \left(Sp_j \cdot (1 - Sp_k) \cdot (1 - p)\right)}$$

Code available at:
https://github.com/EPPIcenter/bolinas-analysis/blob/master/2B_Ab_prevalence_joint_multinomial.R
